# *In utero* and lactational exposure to the Selective Serotonin Reuptake Inhibitor fluoxetine compromises pup bones at weaning

**DOI:** 10.1038/s41598-018-36497-8

**Published:** 2019-01-18

**Authors:** Samantha R. Weaver, Cynthia Xie, Julia F. Charles, Laura L. Hernandez

**Affiliations:** 1 Department of Dairy Science, University of Wisconsin-Madison, Madison, WI USA; 2 Departments of Orthopedics and Medicine, Brigham and Women’s Hospital, Boston, MA USA

## Abstract

Selective Serotonin Reuptake Inhibitors (SSRIs) such as fluoxetine are widely prescribed to pregnant and breastfeeding women, yet the effects of peripartum SSRI exposure on neonatal bone are not known. In adult populations, SSRI use is associated with compromised bone health, and infants exposed to *in utero* SSRIs have a smaller head circumference and are shorter, suggesting possible effects on longitudinal growth. Yet no study to date has examined the effects of peripartum SSRIs on long bone growth or mass. We used microCT to determine the outcomes of *in utero* and lactational SSRI exposure on C57BL6 pup bone microarchitecture. We found that peripartum exposure to 20 mg/kg fluoxetine reduced femoral bone mineral density and bone volume fraction, negatively impacted trabecular and cortical parameters, and resulted in shorter femurs on postnatal day 21. Although SSRIs are considered the first-choice antidepressant for pregnant and lactating women due to a low side effect profile, SSRI exposure may compromise fetal and neonatal bone development.

## Introduction

Although approximately 5% of women reported using a Selective Serotonin Reuptake Inhibitor (SSRI) antidepressant during pregnancy between 1998 to 2005^1^, the effects of SSRI exposure on the infant are not well-defined. SSRI-exposed infants have detectable levels of antidepressant in their blood due to placental and breast milk transfer^2,3^. In adults, SSRI exposure has been associated with low bone mass and increased fracture risk^4,5^, and we have determined that SSRI exposure during pregnancy and lactation reduces maternal trabecular bone mass at 3 months and 9 months post-partum in a rodent model^6^. Additionally, infants exposed to SSRIs are shorter and have a smaller head circumference^7–9^. While growing mice given an SSRI were shown to have reduced bone mineral accrual^10^, no study to date has examined the effects of peripartum SSRI exposure on neonatal long bone development.

To test the hypothesis that *in utero* and lactational SSRI exposure detrimentally affects neonatal bone, we exposed C57BL/6 dams to 20 mg/kg fluoxetine (a commonly prescribed SSRI) from the day of conception through postnatal day 21, at which time pups were euthanized and the femur was harvested. We found that pups exposed to peripartum fluoxetine have shorter femurs and compromised trabecular and cortical tissue bone density and microarchitecture compared to control pups. Although SSRIs are considered the safest antidepressant during pregnancy and lactation, peripartum exposure to the SSRI fluoxetine may compromise neonatal skeletal health.

## Results

### Peripartum fluoxetine exposure impairs trabecular and cortical bone in offspring

**Figure 1:**
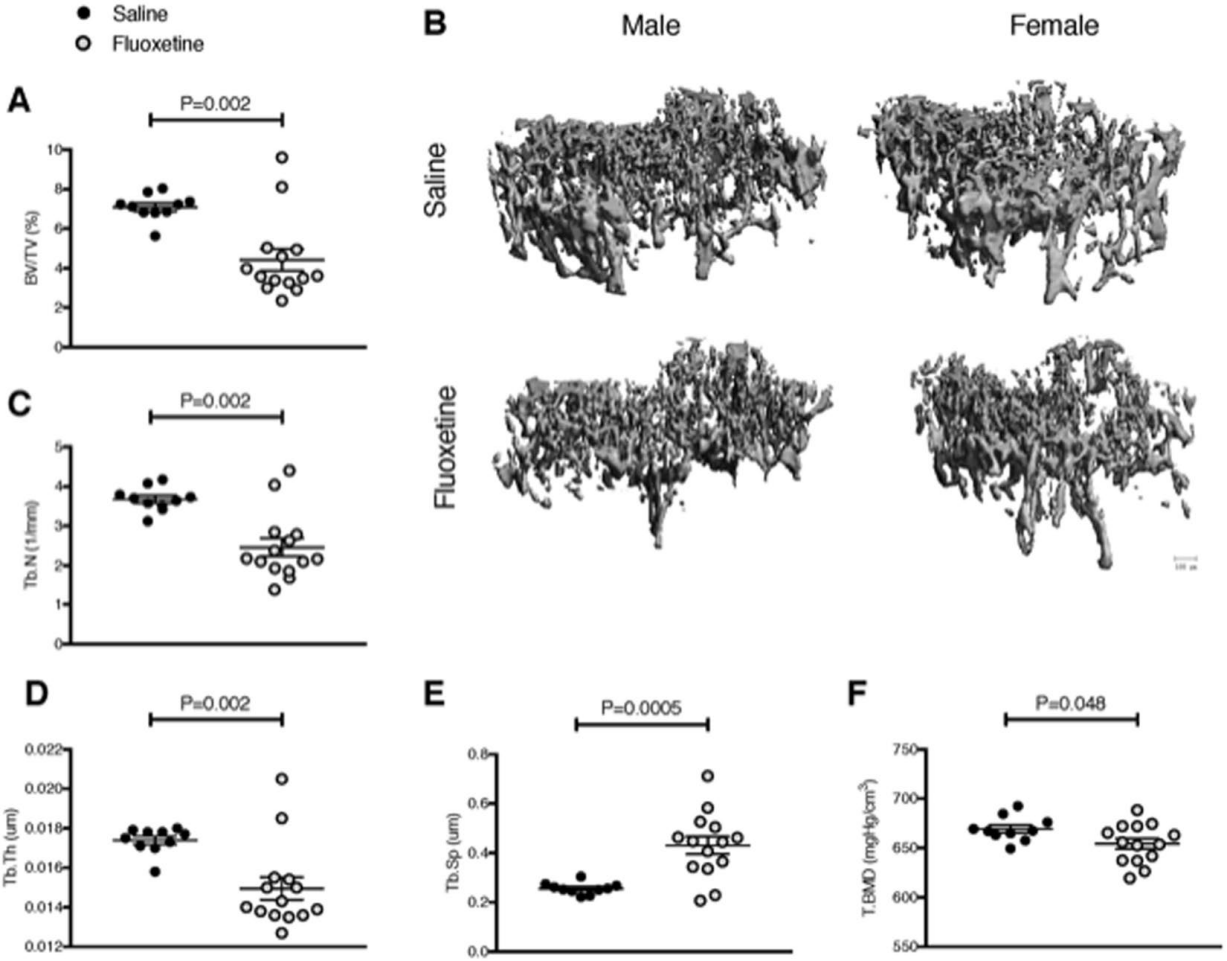
Pups exposed to *in utero* and lactational fluoxetine have compromised trabecular femoral bone. Dams were injected intraperitoneally with either saline or 20 mg/kg fluoxetine from day 0 of pregnancy through day 21 of lactation. At weaning, femurs were extracted from *n* = 4 and *n* = 8 female pups of saline dams and *n* = 6 male and *n* = 8 female pups of fluoxetine dams and subjected to microCT analysis. (**A**) Bone volume/tissue volume. (**B**) 3D reconstruction images of distal femur trabecular bone. The scale bar represents 100 microns. (**C**) Trabecular number (mm^−1^). (**D**) Trabecular thickness (μm). (**E**) Trabecular spacing (μm). (**F**) Trabecular bone mineral density (mgHg/cm^3^). Data were analyzed with a Student’s t-test and are presented as mean ± SEM.

We first confirmed that exposure to fluoxetine *in utero* and throughout lactation increased concentrations of fluoxetine in the pup serum taken on postnatal day 21 (0 vs 5.86 ± 0.8 ng/mL for control and fluoxetine pups, respectively; P = 0.0003). Serum serotonin concentrations on postnatal day 21 were elevated in pups exposed to *in utero* and lactational fluoxetine (2139 ± 196 vs 2800 ± 179 ng/mL for control and fluoxetine pups, respectively; P = 0.02). Due to the known relationship between SSRI use and reduced bone mineral density, we examined the effects of *in utero* and lactational exposure to fluoxetine on trabecular and cortical bone in the femur by microCT. There was no difference in any of the parameters due to sex of the pups (P > 0.05), so data from all pups were pooled. Trabecular bone volume/tissue volume (BV/TV) was lower in pups exposed to peripartum fluoxetine (7.1 ± 0.21 vs 4.1 ± 0.55%; P = 0.002; Fig. 1A,B). Consistent with reduced BV/TV, pups exposed to fluoxetine also had fewer trabeculae (3.7 ± 0.1 vs 2.5 ± 0.2 mm^−1^; P = 0.002; Fig. 1C), thinner trabeculae (0.02 ± 0.0002 vs 0.01 ± 0.0005 μm; P = 0.002; Fig. 1D), and greater trabecular spacing (0.26 ± 0.007 vs 0.43 ± 0.04 μm; P = 0.0005; Fig. 1E) than pups exposed to saline. Additionally, trabecular tissue bone mineral density (BMD) was reduced in pups exposed to fluoxetine (669 ± 4 vs 654 ± 5 mgHg/cm^3^ for control and fluoxetine, respectively; P = 0.048; Fig. 1F).

**Figure 2:**
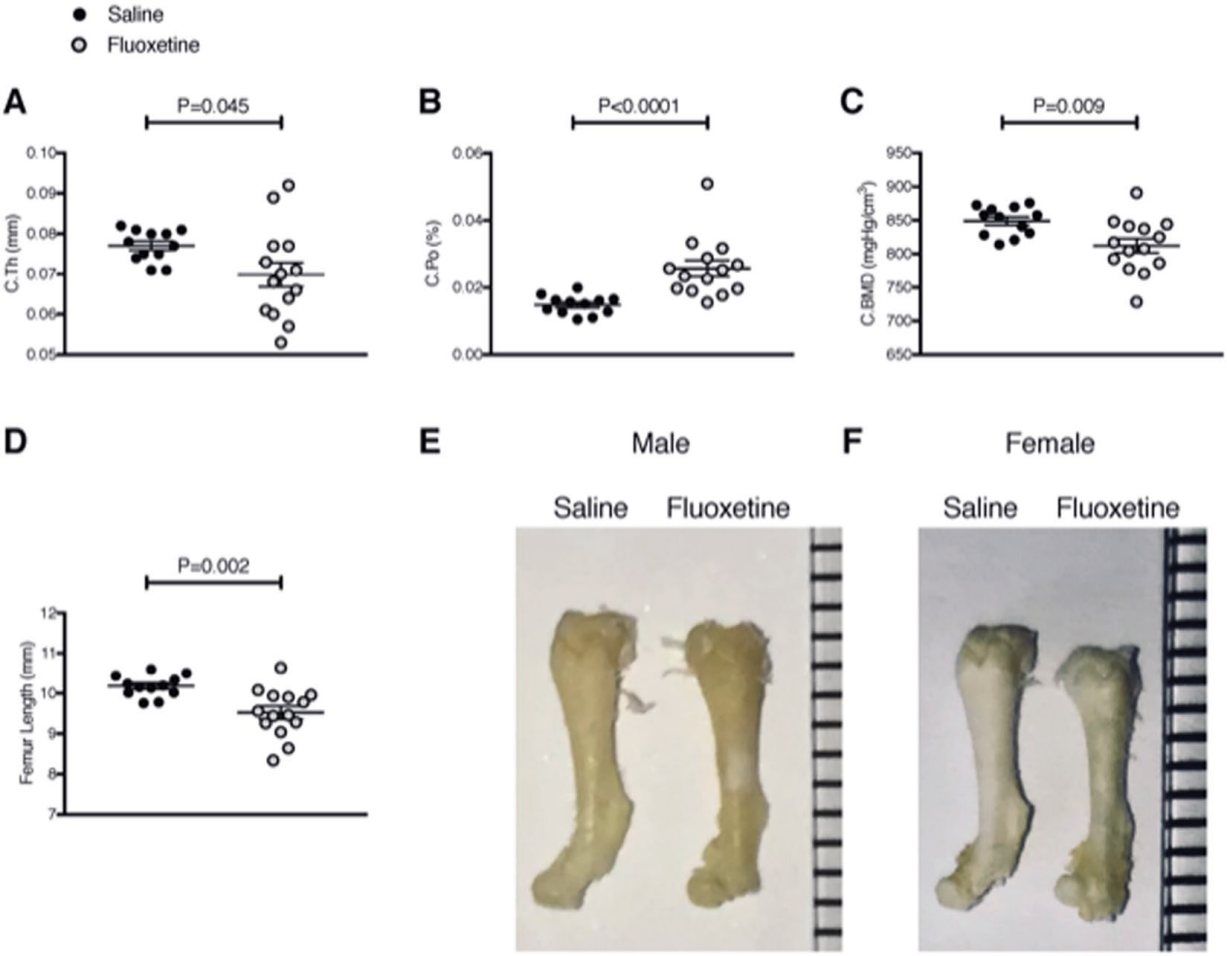
Pups exposed to *in utero* and lactational fluoxetine have compromised cortical femoral bone and shorter femurs. Dams were injected intraperitoneally with either saline or 20 mg/kg fluoxetine from day 0 of pregnancy through day 21 of lactation. At weaning, femurs were extracted from *n* = 4 and *n* = 8 female pups of saline dams and *n* = 6 male and *n* = 8 female pups of fluoxetine dams and subjected to microCT analysis. (**A**) Cortical thickness (mm). (**B**) Cortical porosity (%). (**C**) Cortical bone mineral density (mgHg/cm^3^). (**D**) Femur length (mm). (**E**,**F**) Isolated femurs from saline-treated (left) and fluoxetine-treated (right) mice are shown. Scale bar in mm. Males are shown in (**E**) and females are shown in (**F**). Data were analyzed with a Student’s t-test and are presented as mean ± SEM.

Femoral cortical bone was also detrimentally affected by peripartum fluoxetine exposure. Pups exposed to *in utero* and lactational fluoxetine had thinner cortical bone (0.08 ± 0.001 vs 0.07 ± 0.003; P = 0.045; Fig. 2A), as well as more porous cortical bone (0.01 ± 0.0008 vs 0.03 ± 0.002; P < 0.0001; Fig. 2B). Cortical tissue BMD was also reduced in pups exposed to fluoxetine (849 ± 6.2 vs 812 ± 11 mgHg/cm^3^; P = 0.009; Fig. 2C). Finally, the femurs of pups exposed to fluoxetine were shorter (10.2 ± 0.08 vs 9.5 ± 0.16; P = 0.002; Fig. 2D,E) compared to pups exposed to saline. There was no effect of fluoxetine exposure on endosteal area (P = 0.49), periosteal circumference (P = 0.90), or periosteal area (P = 0.88).

## Discussion

SSRIs are commonly prescribed during pregnancy and lactation as they have fewer adverse side effects on both mother and infant than other antidepressants^11,12^. While SSRI exposure has been associated with increased risk of osteoporosis and fracture in animal studies and human populations^4,5,10^, our report demonstrates that peripartum exposure to fluoxetine compromises femoral bone development in the offspring. There are numerous reports on the neuropsychiatric outcomes in infants exposed to SSRIs, but few studies have examined fetal and infant skeletal development^12–14^. We find that pups exposed to fluoxetine throughout pregnancy and lactation have reduced trabecular and cortical tissue BMD, reduced trabecular BV/TV, as well as thinner and fewer trabeculae and thinner and more porous cortical bone. Moreover, shorter femur lengths in fluoxetine-exposed pups suggest that SSRIs may affect longitudinal bone growth. Collectively, these data demonstrate that peripartal fluoxetine exposure to the dam may have adverse effects on neonatal bone.

Consistent with our finding of a detrimental impact on developing long bone, fluoxetine exposure to pregnant and lactating rats inhibited mandibular development in pups^15^ and reduced whole-body and hindlimb bone mineral accrual in young, growing pups^10^. By the time of parturition, a fetus has formed 98% of its skeleton^16^. However, longitudinal appositional growth continues until puberty in humans^17^ and past sexual maturity in mice^18^. Bone mass also continues to accrue for some time in both species. In future research, it will be important to determine whether the effects of peripartum fluoxetine exposure on mouse femur length and bone mass persist into adulthood or are compensated for during post-weaning growth. Additionally, while we did not detect differences between male and female pups in bone mass or response to fluoxetine exposure on postnatal day 21, future work should examine the effects of fluoxetine in pubertal or post-pubertal models, as hormones are known to regulate bone development^19^.

Components of the serotonin signaling pathway are present on osteoclasts, osteoblasts, and osteocytes^20^. However, there is little consensus on the role of serotonin in the regulation of bone mass^21–23^. Pups exposed to fluoxetine throughout pregnancy and lactation had elevated concentrations of serum serotonin compared to control pups. Exposure to SSRIs typically reduces serum serotonin concentrations^24,25^. Indeed, serum serotonin concentrations were reduced in dams exposed to fluoxetine throughout pregnancy and lactation who gave birth to the pups included in this study^6^. In human populations, cord blood from SSRI-exposed infants has lower concentrations of the serotonin metabolite 5-hydroxyindoleacetic acid compared to control infants^26^. However, studies of breastfeeding mother-infant pairs have shown that exposure to various SSRIs, including fluoxetine, through the breastmilk does not affect infant blood serotonin concentrations^24,25^. It is unclear why pups exposed to fluoxetine throughout pregnancy and lactation had increased concentrations of serum serotonin compared to pups of saline-exposed dams. Importantly, several reports suggest that serotonin promotes bone resorption and inhibits osteoblast formation^27–29^. As such, it is possible that elevated serum serotonin concentrations in fluoxetine-exposed pups acted directly on bone to compromise skeletal development.

Gestational and lactational exposure to fluoxetine reduced bone mineralization, trabecular bone volume fraction, and femoral length in mice. Future research should explore the effects of different classes of SSRIs, as well as varying doses of SSRIs during pregnancy and lactation, on skeletal health of the offspring. Future work would also benefit from a greater number of pups, as well as different methods of SSRI administration, as intraperitoneal injection during murine pregnancy is not ideal. The impact of SSRIs on neonatal bone acquisition and growth, and the possibility of negative consequences for skeletal health later in life, should be explored further in rodent models as well as in human populations.

## Methods

### Animals

All experiments were performed under protocol number A01473 and approved by the Research Animal Care and Use Committee at the University of Wisconsin-Madison. Female C57BL/6 mice were individually housed in a controlled environmental facility for biological research in the Animal Science Department vivarium at the University of Wisconsin-Madison. Mice were either obtained through our mating colony or ordered from Jackson Laboratories when they were between seven to nine weeks of age ± three days (stock 000664, Jackson Laboratories, Bar Harbor, ME). Mice were maintained at a temperature of 25 °C and humidity of 50–60% on a 12-h light/dark cycle with free access to food (Teklad global 19% protein extruded, Envigo 2019) and water. Beginning at seven weeks of age, female mice were bred overnight with a male of approximately the same age. Pregnancy was determined via visualization of the vaginal plug, at which time dams were individually housed. Dams were randomly assigned to two treatments: saline (*n* = 2) or the SSRI fluoxetine (*n* = 4) injections. Beginning on the plug date, dams received daily intraperitoneal injections of either sterile saline or 20 mg/kg bodyweight fluoxetine hydrochloride (Sigma-Aldrich F312) reconstituted in sterile saline. This dose produces plasma fluoxetine concentrations in mice that correspond to humans prescribed 20 to 80 mg/day^30^ and has previously been shown to affect bone tissue in rodent models^31^. The final volume injected into each dam was 0.12 mL of either saline or fluoxetine reconstituted in saline. Injections occurred daily between 0700 h and 0800 h every day from the plug date through day 21 of lactation. We have previously performed intraperitoneal injections in pregnant and lactating dams, and intraperitoneal injections are common among many other studies examining the effects of SSRI^31–34^. To additionally control for the potential stress of injection, control animals were given sham injections. Litters were not standardized due to variation in pup mortality between dams in our colony^6^.

On day 21 of lactation, pups were weaned from the dams and euthanized via carbon dioxide inhalation. One femur was extracted from each pup and whole blood was collected via cardiac puncture. Femurs were extracted from *n* = 4 male and *n* = 8 female saline pups and *n* = 6 male and *n* = 8 female fluoxetine pups (Table 1). Sex-matched pups were selected from each dam whenever possible.

**Table 1:** Distribution of pups exposed to gestational and lactational saline or fluoxetine via intraperitoneal injections to dams.

Dam	Treatment	Female Pups	Male Pups
1	Saline	4	3
2	Saline	4	1
3	Fluoxetine	2	0
4	Fluoxetine	4	2
5	Fluoxetine	0	2
6	Fluoxetine	2	2

### Sample Collection

Blood was collected into Capillary Blood Collection tubes (Terumo T-MG) via cardiac puncture at the time of euthanasia on day 21 of lactation. Blood was allowed to sit at 4 °C overnight in order to allow platelets to disrupt and was then spun down at 3,000 rpm for 20 minutes in order to isolate serum, which was stored at −80 °C until the time of assay.

### Micro-computed tomography (microCT)

Femurs were scanned using a uCT 35 scanner (Scanco Medical) in 70% ethanol. Femur length was digitally measured on scout images. Samples were scanned at isotropic voxel size of 7 microns, with an X-ray tube potential of 55 kVp, an X-ray intensity of 0.145 mA and an integration time of 600 ms. For the scans of the femur, a region beginning 0.1 mm proximal to the growth plate and extending 0.56 mm proximally was selected for trabecular bone analysis. A second region 0.4 mm in length and centered at the midpoint of the femur was used to calculate diaphyseal parameters. A semi-automated contouring approach was used to distinguish cortical from trabecular bone. The region of interest was thresholded using a global threshold that set the bone/marrow cut-off at 584 mg HA/cm^3^ for both trabecular bone and cortical bone. 3-D microstructural properties of bone were calculated using software supplied by the manufacturer and reported according to consensus guidelines on rodent microCT. One control male and one control female were omitted from trabecular analyses because their femurs proximal to the growth plate were damaged while harvesting the bone.

### Assays

Serum was diluted 1:5 and fluoxetine concentrations were determined using Neogen Fluoxetine Test Kit (#107619). Serum serotonin concentrations were determined using a Beckman Coulter Enzyme Immunoassay kit (catalog no. IM1749; Beckman Coulter, Vršovice, Czech Republic). Serum was diluted 1:100 to fit within the standard curve. All tests were performed per the manufacturer’s instructions.

### Statistical analysis

All statistics were conducted using Prism Graphpad (Version 7.0c for Mac OS X). Data was first checked for normality using a Pearson normality test. Normal data was analyzed using an unpaired Student’s two-sided t-test. When the normality assumption failed, data was analyzed using a Mann-Whitney for non-parametric data. Differences between means were considered significant at P < 0.05. All values are reported as means ± standard error of the mean (SEM).

## Data Availability

The datasets generated during and/or analyzed during the current study are available from the corresponding author on reasonable request.
